# In Silico Screening of 1,3,4-Thiadiazole Derivatives as Inhibitors of Vascular Endothelial Growth Factor Receptor-2 (VEGFR-2)

**DOI:** 10.3390/cimb46100666

**Published:** 2024-10-06

**Authors:** Steven M. Ewell, Hannah Burton, Bereket Mochona

**Affiliations:** 1 Department of Chemistry, Florida A&M University, Tallahassee, FL 32307, USA; steven1.ewell@famu.edu; 2 College of Pharmacy and Pharmaceutical Sciences, Institute of Public Health, Florida A&M University, Tallahassee, FL 32307, USA

**Keywords:** molecular docking, molecular dynamics simulation, virtual screening, Computer-Aided Drug Design (CADD), receptor tyrosine kinases (RTKs)

## Abstract

Angiogenesis plays a pivotal role in the growth, survival, and metastasis of solid tumors, with Vascular Endothelial Growth Factor Receptor-2 (VEGFR-2) being overexpressed in many human solid tumors, making it an appealing target for anti-cancer therapies. This study aimed to identify potential lead compounds with azole moiety exhibiting VEGFR-2 inhibitory effects. A ligand-based pharmacophore model was constructed using the X-ray crystallographic structure of VEGFR-2 complexed with tivozanib (PDB ID: 4ASE) to screen the ZINC15 database. Following virtual screening, six compounds demonstrated promising docking scores and drug-likeness comparable to tivozanib. These hits underwent detailed pharmacokinetic analysis to assess their absorption, distribution, metabolism, excretion, and toxicity (ADMET) properties. Furthermore, Density Functional Theory (DFT) analysis was employed to investigate the molecular orbital properties of the top hits from molecular docking. Molecular dynamics (MD) simulations were conducted to evaluate the conformational stability of the complexes over a 100 ns run. Results indicated that the compounds (ZINC8914312, ZINC8739578, ZINC8927502, and ZINC17138581) exhibited the most promising lead requirements for inhibiting VEGFR-2 and suppressing angiogenesis in cancer therapy. This integrated approach, combining pharmacophore modeling, molecular docking, ADMET studies, DFT analysis, and MD simulations, provides valuable insights into the identification of potential anti-cancer agents targeting VEGFR-2.

## 1. Introduction

Despite the development of many anti-cancer drugs over the years, cancer remains a prominent cause of death worldwide. Cancer therapy interfering with a single biological molecule or pathway has been successfully utilized. However, significant concerns related to selectivity, pharmacokinetic properties, mutation resistance, and safety have been raised [1,2,3]. Thus, there is an urgent need to develop specific agents using various means of drug discovery. Computer-Aided Drug Design (CADD) in lead discovery and optimization, such as pharmacophore modeling, virtual screening, molecular docking, and molecular simulation methods, expedite the discovery and evaluation of potential therapeutic agents [4,5].

Receptor tyrosine kinases (RTKs) constitute a family of cell surface receptors that regulate various cellular processes, including proliferation, differentiation, and migration, through the activation of intracellular signaling pathways [6,7]. Angiogenesis heavily relies on RTK-mediated signaling, particularly the VEGF signaling pathway. Vascular endothelial growth factors (VEGFs) bind to their cognate receptors, primarily VEGFR-1 (Flt-1) and VEGFR-2 (KDR/Flk-1), initiating a cascade of events that promote endothelial cell proliferation, migration, and vessel formation [8,9]. Among these receptors, VEGFR-2 stands out as the principal mediator of VEGF-induced angiogenesis, making it an attractive target for anti-angiogenic therapies.

VEGFR-2, also known as kinase, insert domain receptor (KDR), or fetal liver kinase-1 (Flk-1), is a transmembrane RTK predominantly expressed on endothelial cells. Activation of VEGFR-2 by VEGF ligands leads to receptor dimerization, autophosphorylation, and subsequent activation of downstream signaling pathways, including the phosphoinositide 3-kinase (PI3K)/AKT and mitogen-activated protein kinase (MAPK) pathways [4,5,6]. These signaling cascades orchestrate endothelial cell proliferation, survival, and migration, ultimately culminating in the formation of new blood vessels. Given its pivotal role in angiogenesis, the dysregulation of VEGFR-2 signaling is implicated in various pathological conditions, particularly cancer, where excessive angiogenesis fuels tumor growth and metastasis [10,11,12].

The advent of computational techniques has revolutionized drug discovery and development, particularly in the design of targeted therapies against specific molecular targets. CADD encompasses a range of computational methods, including molecular modeling, virtual screening, and pharmacophore modeling, to expedite the identification and optimization of lead compounds with desired pharmacological properties. In the context of angiogenesis inhibition, CADD holds immense potential for rational drug design targeting VEGFR-2, enabling the rapid screening of chemical libraries and prediction of ligand-receptor interactions [13,14].

Tivozanib (Fotivda™) is a potent and selective inhibitor of VEGFR-1, -2, and -3, FDA-approved for the treatment of advanced renal cell carcinoma (RCC). Its high specificity for VEGFR-2 inhibition makes it an attractive reference compound for studying VEGFR-2-targeted therapies. Tivozanib exerts its anti-angiogenic effects by binding to the ATP-binding site of VEGFR-2, preventing receptor phosphorylation and downstream signaling [13,14]. Preclinical and clinical studies have demonstrated the efficacy of Tivozanib in inhibiting tumor angiogenesis and delaying disease progression in RCC patients, highlighting its therapeutic potential as a VEGFR-2 inhibitor [15,16,17].

In recent years, five-membered azole rings have garnered significant attention in drug design and development, particularly in the context of VEGFR-2 inhibition and anti-angiogenic therapy. Azole-containing compounds, such as imidazoles and triazoles, serve as versatile pharmacophores due to their ability to chelate metal ions and form hydrogen bonds with target proteins [18,19]. These heterocyclic motifs often serve as key structural elements in small molecule inhibitors, including those targeting VEGFR-2, by occupying the ATP-binding site and disrupting kinase activity. Moreover, the unique electronic properties of azole rings contribute to their potency and selectivity, making them attractive scaffolds for the design of novel VEGFR-2 inhibitors with improved pharmacological profiles. Incorporating five-membered azole rings into the molecular framework of VEGFR-2 inhibitors represents a promising strategy in computer-aided drug design, aiming to enhance potency, selectivity, and drug-like properties while minimizing off-target effects. Therefore, the exploration of five-membered azole rings holds great potential in advancing the development of next-generation anti-angiogenic agents for cancer therapy, synergizing with the computational approaches employed in drug discovery pipelines [20,21,22].

## 2. Result and Discussion

### 2.1. Pharmacophore Modeling

Pharmacophore modeling plays a crucial role in the realm of drug discovery and design by aiding in the pinpointing of small molecule ligands that specifically bind to target proteins. Fundamentally, a pharmacophore encapsulates the fundamental characteristics of a ligand necessary for its interaction with the target protein and consequent biological response. These characteristics encompass hydrogen bond donors and acceptors, hydrophobic regions, aromatic rings, as well as positively or negatively charged groups. By unraveling the pharmacophore of a target protein, researchers can meticulously screen compound libraries to identify potential drug candidates possessing optimal binding properties [23,24,25].

**Figure 1:**
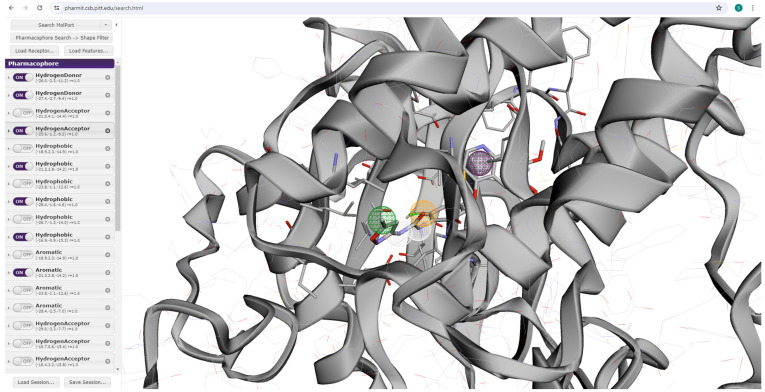
Screening of Phamit database via pharmacophore.

Using Pharmit servers, we initiated the screening of a comprehensive compound database to identify potential drug candidates. By applying specific pharmacophoric features such as hydrogen donors, hydrogen acceptors, hydrophobic regions, and aromatic rings, we meticulously constructed a pharmacophore model. Each feature’s spatial coordinates, along with its respective vectors and radii, were precisely defined to capture the essential interaction points necessary for binding efficacy. This model served as the template for our virtual screening, ensuring that only compounds matching the defined pharmacophoric criteria were considered. The pharmacophoric criteria (Figure 1) for the model (PDB ID:4ASE) were established based on key interactions observed in the protein–ligand complex. Hydrogen bond donors were identified as the nitrogen atoms from the diamine linkage, while the hydrogen bond acceptor was the oxygen atom from the same linkage, as these groups form three crucial hydrogen bonds with the protein. Hydrophobic regions were characterized by the terminal ends of the molecule and the ring system, contributing to essential hydrophobic interactions. The aromatic ring was selected for its role in additional hydrogen bonding interactions within the binding site.

The pharmacophore model (Figure 1) was generated utilizing the Pharmit web server, utilizing the crystallographic structure of Vascular Endothelial Growth Factor Receptor 2 (VEGFR2) complexed with Tivozanib (PDB ID:4ASE). Pharmit conducted a comprehensive search for prospective ligand features within the pharmacophore hypothesis to be recognized as potential VEGFR-2 inhibitor candidates. A total of 62 molecules were sourced from the Pharmit for further in-depth analyses.

### 2.2. Molecular Docking

Molecular docking stands out as a crucial and extensively utilized technique in low-cost computer-assisted drug design. At its heart, molecular docking involves positioning small molecules within the active site of the target enzyme. This process utilizes scoring functions to gauge a compound’s potential biological activity, aiding in the prediction of ligand accessibility within a biological context. By exploring three-dimensional structural arrangements, molecular docking unveils valuable insights into how a specific ligand might interact with different regions of its target protein, all elucidated through the scoring functions [18,26].

**Figure 2:**
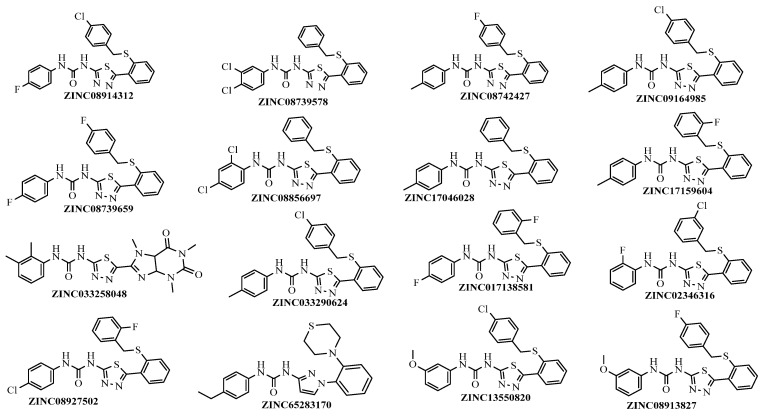
Structure 16 top compounds from screening Pharmit.

All 62 molecules docked with the RTK VEGFR-2 (4ASE) to assess their binding affinities. Among the compounds evaluated, Tivozanib, the reference compound, achieved the lowest docking score of −12.135 kcal/mol, indicating the strongest binding affinity among all tested molecules. In this study, a binding affinity threshold of −8.0 kcal/mol was utilized as a criterion for high affinity, selecting compounds with a strong potential for interaction with the target. Based on this criterion, the top 16 compounds (Table 1) exhibiting binding affinities equal to or lower than −8.0 kcal/mol were identified and further analyzed for their therapeutic potential. The top 16 compounds (Figure 2) have been selected for further experimental validation to assess their potential as drug candidates. The 16 compounds were in the active site similarly to the (Figures S1 and S2).

The molecular docking analysis revealed that most ZINC compounds demonstrated hydrogen bond interactions with GLU885 and ASP1046 via the diamine linkage, a critical aspect of their binding affinity and stability within the active site. Notably, ZINC08856697 exhibited a distinct interaction with ASP1051, facilitated by the chlorine group on the ligand, indicating a unique binding orientation. ZINC00008927502 also showed interactions with GLU885 and ASP1046 but through a different functional group, suggesting alternative binding modes that might influence its efficacy and specificity (Figures S3–S17).

**Figure 3:**
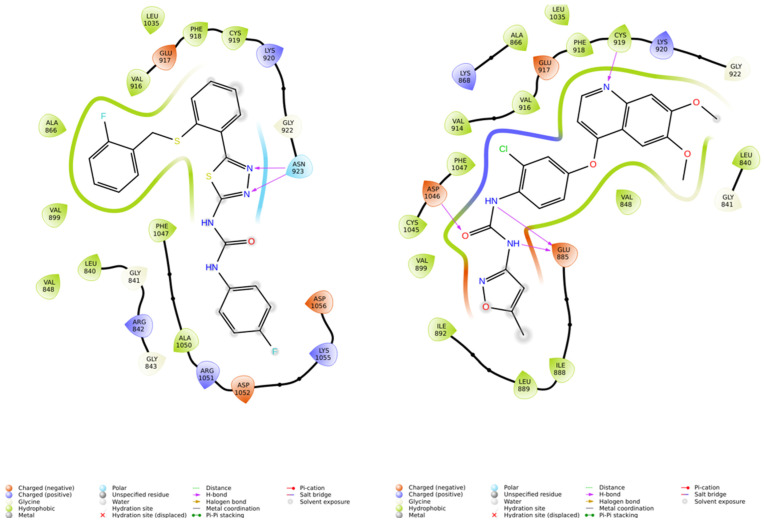
Ligand interaction of the 4ASE complexes of ZINC000017138581 (**left**) and Tivozanib (**right**).

ASP1051, GLU885, ASP1046, and ASN923 represent key amino acids involved in the interaction between 4ASE and Tivozanib. GLU885 and ASP1046 are critical as they form three hydrogen bonds with Tivozanib, significantly contributing to its binding affinity. This interaction is crucial because it stabilizes the ligand within the binding site, enhancing its affinity and specificity for the target. Most of the 16 compounds identified in this study also interact with GLU885 and ASP1046, indicating that these amino acids play a pivotal role in binding. However, a few compounds only form hydrogen bonds with ASP1051 and ASN923, highlighting that while these interactions are less comprehensive, they still contribute to the binding process. Hydrogen bonding is a fundamental aspect of molecular docking as it directly affects the stability and specificity of the ligand–protein complex, influencing the overall docking score and the potential efficacy of the compounds [17,18,19,20,21,22]. Compounds ZINC000017138581 and Tivozanib displayed two hydrogen bonds with ASN923, the nitrogen–nitrogen group on the 1,3,4-thiadiazole moiety, highlighting this group’s potential role in enhancing binding affinity (Figure 3). ZINC33258048 had a docking score of −8.630 kcal/mol, reflecting a moderately strong interaction, while ZINC000017138581 and ZINC000008927502 had scores of −8.520 kcal/mol and −8.286 kcal/mol, respectively.

### 2.3. ADMET Studies

ADMET studies are pivotal in drug discovery and development, encompassing absorption, distribution, metabolism, excretion, and toxicity assessments. These investigations scrutinize how potential drug candidates behave within the human body, evaluating their absorption into the bloodstream, distribution to target tissues, metabolism by enzymes, elimination from the body, and any potential toxicity concerns [22,27]. These criteria are grounded in the understanding that compounds failing to meet these thresholds may encounter challenges in membrane permeability, oral absorption, and pharmacokinetic performance. ADMET studies thus serve to validate a compound’s interactions with biological systems and inform decision-making in the drug development pipeline [28,29].

All the compounds listed fall within or are close to the acceptable molecular weight range for drug-like molecules (Table 2). Higher MW can sometimes indicate complexity in synthesis and potential issues with cell permeability, but all values here are within a manageable range. The number of rotatable bonds (#rotor) for all compounds is moderate (4 to 6), indicating flexibility without excessive molecular complexity that could hinder oral bioavailability. The dipole moments of these compounds, which range from 3.229 to 9.84, indicate the polar nature of these molecules. The solvent-accessible surface area SASA values span from 726.902 to 789.923, indicating the compounds’ potential surface exposure to solvent, which is crucial for solubility and interaction with the biological environment. FOSA and FISA provide insights into the balance between hydrophobic and hydrophilic regions, which is critical for membrane permeability and solubility, reflecting significant π interactions, which are vital for binding to aromatic amino acids in proteins. WPSA indicates substantial weakly polar surface areas, potentially impacting its interaction with biological molecules.

Among the ZINC database compounds, ZINC000008914312 and ZINC000008739578 emerge as strong candidates (Table 3). Both compounds demonstrate excellent Caco-2 permeability (QPP-Caco values of 593.714 and 563.062, respectively), indicative of high intestinal absorption. Their moderate QPlogPo/w values of 5.252 and 5.591, respectively, suggest sufficient lipophilicity for effective membrane permeability without excessive hydrophobicity that could lead to poor solubility. Despite their high QPlogHERG values (−5.712 and −6.047, respectively), which raise potential cardiotoxicity concerns, their overall profiles are favorable. ZINC000008739578, in particular, shows a relatively balanced QPlogBB value of −0.564, indicating the potential for adequate brain penetration, which can be advantageous for targeting brain tumors. Additionally, both compounds adhere to the Veber Rule, with acceptable numbers of rotatable bonds and PSA values.

ZINC000017138581 has a moderate lipophilicity (QPlogPo/w of 4.872) but poor solubility (QPlogS of −6.794) and potential cardiotoxicity (QPlogHERG of −5.79), violating Jorgensen’s Rule of Three. However, it shows good intestinal permeability (QPP-Caco of 581.815), suggesting efficient absorption. ZINC000008927502 presents both promising attributes and notable concerns regarding its potential as a drug candidate. This compound demonstrates a QPlogPw of 13.606 and a QPlogPo/w of 5.579, indicating moderate lipophilicity conducive to membrane permeability. However, its predicted aqueous solubility (QPlogS of −8.061) and conformation-independent solubility (CIQPlogS of −7.645) fall outside the acceptable range defined by Jorgensen’s Rule of Three, suggesting poor solubility that could hinder bioavailability.

Moreover, ZINC000008927502 has a QPlogHERG value of −6.544, indicating a significant risk for cardiotoxicity due to potential HERG K+ channel blockade, which raises safety concerns. The compound also complies with the Veber Rule, exhibiting an appropriate number of rotatable bonds and a polar surface area (TPSA) that suggests favorable pharmacokinetic properties. Additionally, the QPP-Caco value of 570.418 indicates good permeability, supporting the potential for effective oral absorption.

Overall, ZINC000008914312, ZINC000008739578, and ZINC000017138581 are identified as the most promising compounds for further investigation and optimization in the development of anti-cancer drugs, considering their balanced physicochemical properties and potential for high oral bioavailability.

### 2.4. Density Functional Theory

Density Functional Theory (DFT) serves as a foundational tool in molecular analysis, particularly in studying charge distribution and molecular behavior. At its core are the Highest Occupied Molecular Orbital (HOMO) and Lowest Unoccupied Molecular Orbital (LUMO), crucial descriptors that provide insights into numerous chemical processes involving electrons. Understanding the characteristics of HOMO and LUMO becomes paramount in this regard. In analyzing a selection of compounds, their energies, E_HOMO_ and E_LUMO_, stand as well-established quantum mechanical parameters significantly influencing various chemical interactions [30,31]. DFT calculations can complement molecular docking and dynamics studies [32]. Another practical method for assessing a molecule’s chemical stability is the Frontier Molecular Orbital (FMO) theory, which focuses on these pivotal orbitals. The energy levels of HOMO and LUMO offer valuable insights into energy distribution within a molecule (Table 4). Compound stability can be evaluated by examining the negative values of E_HOMO_ and E_LUMO_. The crucial energy gap (E_HOMO_–_ELUMO_) emerges as a determining factor in understanding a molecule’s chemical reactivity and kinetic stability, with larger energy gaps indicating enhanced stability [30,31,32].

Among the ZINC compounds, ZINC000017138581 exhibits a similar trend with a HOMO of −0.212671 Hartrees, a LUMO of −0.042962 Hartrees, and an energy gap of −0.169709 Hartrees. This narrow energy gap suggests a promising reactivity profile comparable to Tivozanib. Another notable compound, ZINC33258048, shows a HOMO of −0.215602 Hartrees and a LUMO of −0.046696 Hartrees, resulting in an energy gap of −0.168906 Hartrees, indicating favorable electronic properties for potential therapeutic applications. ZINC000033290624 and ZINC65283170 exhibit significantly smaller energy gaps of −0.147012 and −0.152592 Hartrees, respectively, indicating higher potential reactivity. ZINC000008927502 has a moderate energy gap, suggesting that the compound possesses a stable electronic configuration, potentially indicating lower reactivity, which is favorable for its application in drug design.

### 2.5. Molecular Dynamics Simulation

Molecular dynamics (MD) simulation stands out as the primary method in computer-assisted drug discovery pathways, offering insight into whether designed compounds exhibit biological activity [21]. The core principle of MD simulation involves scrutinizing the physical movement of atoms within a molecule, utilizing intermolecular interactions under dynamic conditions over time. This approach yields ample information, including enzymatic reactions, chemical pathways, thermodynamic and kinetic stability, and more. To identify promising drug candidates, designed compounds must meet all conditions in the MD simulation study, demonstrating their ability to interact with the target protein under biological conditions over time. This process confirms whether a specific compound is biologically stable or unstable, thus facilitating effective drug discovery [24,33].

Based on the results from molecular docking ADMET analyses and DTF calculations, ZINC000008914312, ZINC000008739578, ZINC000008927502, and ZINC000017138581 have been selected for further investigation through molecular dynamics (MD) simulations. These compounds exhibit promising binding interactions, particularly involving the 1,3,4-thiadiazole moiety, which appears to play a crucial role in enhancing their affinity for target proteins. The combination of favorable docking scores and manageable ADMET profiles indicates that these molecules possess significant potential for further development as anti-cancer agents. MD simulations will provide deeper insights into the dynamic behavior and stability of these compounds in a biological context, ultimately guiding optimization efforts for their therapeutic application.

#### 2.5.1. Root Mean Square Deviation (RMSD)

**Figure 4:**
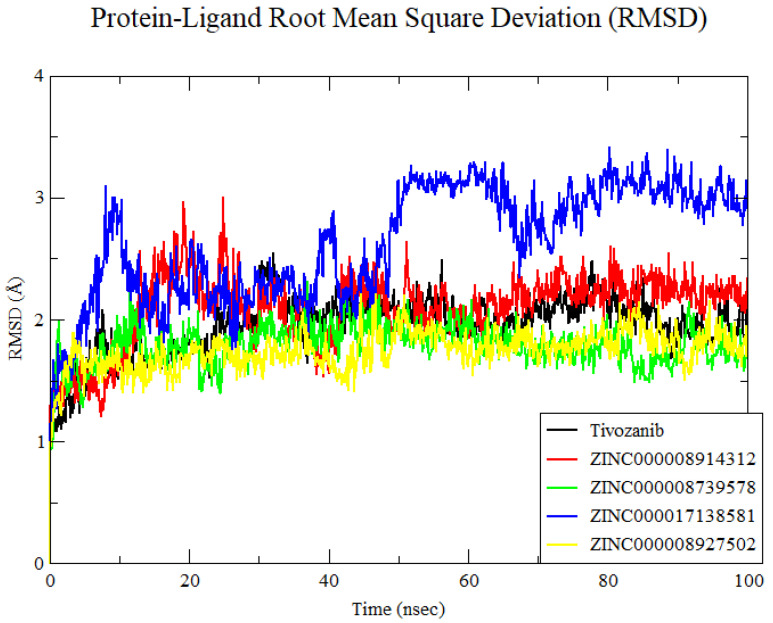
Root Mean Square Deviation of the 4ASE–ligand complexes.

The Root Mean Square Deviation (RMSD) of the Alpha carbon protein for all investigated systems to assess convergence and trajectory stability. The Root Mean Square Deviation (RMSD) analysis of the four ZINC compounds, along with the reference compound, was conducted to assess the stability of the ligand–receptor complexes over a 100 ns molecular dynamics simulation. The RMSD trajectories for ZINC000008914312, ZINC000008739578, and ZINC000008927502, as well as the reference compound, demonstrated consistent stability, maintaining similar conformations throughout the simulation period. For the compound ZINC000008914312 (Figure 4), the observed deviation in the RMSD between 15 and 20 ns likely reflects a conformational adjustment of the ligand–protein complex. This period is within the initial phase of the simulation, where the system stabilizes after the initial conditions have been set. These compounds exhibited minor fluctuations, indicative of stable binding within the active site of the target protein. In contrast, ZINC000017138581 displayed several notable deviations at 40 ns, 50 ns, and 65 ns, suggesting transient conformational changes or potential instability in the binding mode (Figure 4). These deviations may be attributed to the unique structural features of ZINC000017138581, potentially influencing its interaction dynamics with the target protein.

#### 2.5.2. Root Mean Square Fluctuation (RMSF)

**Figure 5:**
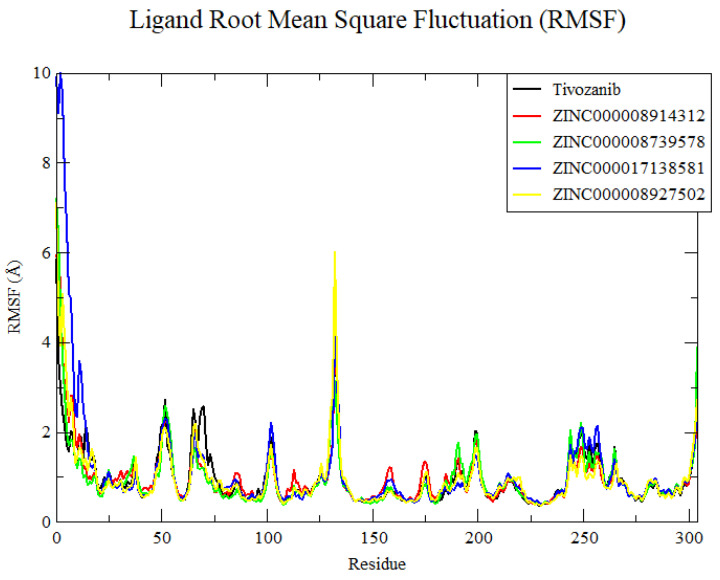
Root mean square fluctuation of the 4ASE–ligand complexes.

The root mean square fluctuation (RMSF) analysis of the four ZINC compounds, along with the reference compound, provided insights into the flexibility and interaction dynamics of the ligand–receptor complexes. The RMSF profiles for ZINC000008914312, ZINC000008739578, ZINC000008927502, and ZINC000017138581 revealed similar interaction patterns, highlighting key residues that contribute significantly to the binding stability (Figure 5). All compounds demonstrated high interactions with GLU885, CYS919, ASN923, ASP1046, and PHE1047 (Figures S18–S22), indicating these residues play a crucial role in maintaining the binding affinity and stability of the complexes. The consistent interaction with these residues suggests a common binding mode among the ZINC compounds and the reference compound, underscoring their potential as viable candidates for further drug development.

### 2.6. Molecular Mechanics Generalized Born Surface Area (MM-GBSA)

To further validate the stability and binding affinity of the selected compounds ZINC000008914312, ZINC000008739578, ZINC000008927502, and ZINC000017138581, we employed the Molecular Mechanics Generalized Born Surface Area (MM-GBSA) approach. This method provides an accurate estimation of the free energy of binding, integrating molecular mechanics energy with solvation effects [34]. By analyzing the MD simulation trajectories, MM-GBSA calculations offer a quantitative assessment of the interaction energies (Table 5), confirming the robustness of the docking results [34,35]. The MM-GBSA results demonstrated consistently favorable binding free energies for all selected compounds, supporting their high affinity and stability within the active site. These findings corroborate the docking studies and underscore the potential of the 1,3,4-thiadiazole moiety in enhancing molecular interactions, reinforcing the viability of these compounds for further optimization and development as anti-cancer therapeutics.

The MM-GBSA calculations provide a detailed assessment of the binding free energies (ΔG Bind) for the selected compounds, offering insights into their potential as anti-cancer agents. The binding free energy for Tivozanib was calculated to be −21.95 kcal/mol, with significant contributions from van der Waals interactions (ΔG Bind vdW of −27.5 kcal/mol) and lipophilic interactions (ΔG Bind Lipo of −7.28 kcal/mol), while the solvation energy (ΔG Bind Solv GB) offset these interactions to some extent (15.7 kcal/mol). For ZINC000008914312, the binding free energy was substantially lower at −58.95 kcal/mol, driven primarily by strong Coulomb interactions (ΔG Bind Coulomb of −16 kcal/mol) and a significant lipophilic component (ΔG Bind Lipo of −23.01 kcal/mol). The solvation energy was high (38.22 kcal/mol), yet the overall binding affinity remained favorable due to the large van der Waals contribution (ΔG Bind vdW of −56.66 kcal/mol).

ZINC000008739578 exhibited an even lower binding free energy of −64.75 kcal/mol, with substantial contributions from both Coulomb interactions (ΔG Bind Coulomb of −11.22 kcal/mol) and lipophilic interactions (ΔG Bind Lipo of −31.96 kcal/mol). The solvation energy was less offsetting (24.45 kcal/mol), leading to a strong overall binding affinity. ZINC000017138581 and ZINC000008927502 showed the most favorable binding free energies of −67.37 kcal/mol and −69.81 kcal/mol, respectively. For ZINC000017138581, the binding was largely influenced by Coulomb interactions (ΔG Bind Coulomb of −19.8 kcal/mol) and lipophilic interactions (ΔG Bind Lipo of −25 kcal/mol), with notable solvation energy (27.78 kcal/mol) being partially offset by van der Waals interactions (ΔG Bind vdW of −53.68 kcal/mol). Similarly, ZINC000008927502’s strong binding affinity was driven by Coulomb interactions (ΔG Bind Coulomb of −19.57 kcal/mol) and lipophilic interactions (ΔG Bind Lipo of −25.78 kcal/mol), with solvation energy (28.35 kcal/mol) balanced by van der Waals interactions (ΔG Bind vdW of −55.99 kcal/mol). Overall, the MM-GBSA results indicate that ZINC000008914312, ZINC000008739578, ZINC000017138581, and ZINC000008927502 exhibit strong binding affinities, primarily driven by favorable Coulomb, lipophilic, and van der Waals interactions, underscoring their potential as promising anti-cancer drug candidates.

## 3. Materials and Methods

### 3.1. Generating Pharmacophore Model

The pharmacophore modeling process utilized in this study involved the utilization of Pharmit (http://pharmit.csb.pitt.edu/, accessed on 1 June 2024), a computational tool designed for pharmacophore-based virtual screening of multiple databases. The 3-D structure of the VEGFR-2 protein (PDB: 4ASE) was loaded into the system as a PDB file from the Protein Data Bank (https://www.rcsb.org/ accessed on 1 June 2024).

### 3.2. Molecular Docking

To conduct standard precision ligand docking using Maestro 2024-01, the procedure begins with ligand preparation, where ligands are processed using LigPrep [35] to generate diverse ionization and tautomeric states. Following this, the protein (4ASE is prepared using Protein Preparation Wizard [36] to optimize hydrogen bonding and minimize steric clashes. A receptor grid with dimensions X = 20.0 Å, Y = 20.0 Å, Z = 20.0 Å is generated around the active site of 4ASE using the Receptor Grid Generation panel, defining a box size adequate to encompass potential ligand binding sites. The grid is customized to include a constriction atom LEU:840, crucial for refining ligand interactions [37]. Finally, standard precision ligand docking is performed within Maestro Ligand Docking module [37], utilizing Glide algorithms to evaluate ligand binding modes and energies within the defined grid, facilitating the identification of potential lead compounds.

### 3.3. ADMET Profiling

The 16 molecules and the reference compound were further subjected to absorption, distribution, metabolism, excretion, and toxicity (ADMET) and drug-likeness evaluation. The ADMET and drug-likeness predictions were performed with QikProp [38]. The molecular structures were first prepared using the LigPrep module within the Schrödinger Suite to ensure proper protonation states and stereochemistry. Subsequently, the prepared structures were subjected to QikProp analysis to evaluate key pharmacokinetic parameters.

### 3.4. DFT Calculations

The 16 molecules and the reference compound were subjected to optimization using Maestro 2024-01. The optimization employed B3LYP theory and a 6–31G** basis set [39] The HOMO and LUMO were computed by closely monitoring molecular surfaces and atomic electrostatic potential charges. The electronic excitation energy was determined. This energy calculation focuses on the HOMO-LUMO gap energy, which is essentially the difference between the HOMO and LUMO energy levels [39,40].

### 3.5. MD Simulation

In this study, molecular dynamics (MD) simulations were prepared complexes using Desmond’s System Builder [41]; the following procedure was followed: First, the system was set up in an orthorhombic box with dimensions of 20.0 Å in each direction, accommodating the solute and solvent molecules. The solvent model chosen was SPC (Simple Point Charge), which is commonly used for water simulations. A salt concentration of 0.15 M NaCl was added to mimic physiological conditions. The simulation parameters were set to run for 100 nanoseconds (ns) using an NPT ensemble with a temperature of 300 K and pressure of 1.2 atm, ensuring constant temperature and pressure throughout the simulation. This setup allows for the investigation of the dynamics and interactions of the complexes over an extended period, providing insights into their stability and behavior in biological environments.

### 3.6. Molecular Mechanics Generalized Born Surface Area (MM-GBSA)

The validation of the binding interactions for the selected compounds ZINC000008914312, ZINC000008739578, ZINC000008927502, and ZINC000017138581 was performed using the MM-GBSA approach with Maestro 2024-01. The simulations were conducted utilizing the Prime module within Maestro, applying the VSGB (Variable Dielectric Surface Generalized Born) solvation model and the OPLS_2005 (Optimized Potentials for Liquid Simulations) force field [42]. The ligand–receptor complexes obtained from molecular docking were subjected to a series of minimization steps to relax the structures prior to the MM-GBSA calculations. The binding free energies were then computed by extracting frames from the MD trajectories and analyzing them with the MM-GBSA method, providing insights into the stability and strength of the molecular interactions within the biological system.

## 4. Conclusions

In conclusion, our study identified four potential drug candidates exhibiting moderate-to-low acute oral toxicity, thus indicating their safety for oral administration. These compounds showed promising binding affinities with the RTK VEGFR-2, as evidenced by computational calculations. Based on an integrated analysis of molecular docking, ADMET properties, DFT calculations, molecular dynamics (MD) simulations, and MM-GBSA binding free energy estimations, the compounds ZINC000008914312, ZINC000008739578, ZINC000008927502, and ZINC000017138581 exhibit promising potential as anti-cancer agents. The molecular docking studies highlighted consistent hydrogen bond interactions with key residues GLU885 and ASP1046, except for ZINC08856697, which interacted with residue 1051, and ZINC000008927502, which formed additional hydrogen bonds with a different moiety. Notably, compounds ZINC000017138581 and ZINC000008927502 displayed two hydrogen bonds with ASN923 through the nitrogen–nitrogen group on the 1,3,4-thiadiazole moiety, highlighting this group’s potential role in enhancing binding affinity.

The ADMET analysis confirmed that these compounds generally adhered to Jorgensen’s Rule of Three and Veber’s rule, indicating favorable pharmacokinetic profiles. DFT studies revealed that the selected compounds possess suitable HOMO-LUMO energy gaps, suggesting optimal electronic properties for stable drug–receptor interactions. MD simulations confirmed the stability of these interactions over time, further validating the initial docking results. Notably, the MM-GBSA calculations showed highly favorable binding free energies, with significant contributions from Coulomb, lipophilic, and van der Waals interactions.

The combined results indicate that the 1,3,4-thiadiazole moiety plays a crucial role in the binding and stability of these compounds, reinforcing their potential as viable candidates for further development in anti-cancer therapy. Our study provides new insights by exploring the interaction of 1,3,4-thiadiazole derivatives, specifically with the VEGFR family of receptor tyrosine kinases (RTKs). RTKs, especially those in the VEGFR family, represent a significant portion of modern drug targets due to their role in angiogenesis and cancer progression. Further experimental validation, including in vitro and in vivo studies, is warranted to confirm their efficacy and safety as anti-cancer medications. Such validation will provide a comprehensive understanding of the pharmacological profile and therapeutic potential of these compounds, paving the way for their development as novel anti-cancer drugs. The design, synthesis, and in vitro and in vivo investigations are underway to identify a new class of thiazole diamide-bearing small molecules as a potential cancer agent.

## Figures and Tables

**Table 1:** Binding affinity scores for 4ASE–Zinc compound and reference molecule.

Molecule	Molecular Formula	Docking Score (kcal/mol)
Tivozanib	C_22_H_19_ClN_4_O_5_	−12.135
ZINC000008914312	C_22_H_16_ClFN_4_OS_2_	−9.036
ZINC000008739578	C_22_H_16_Cl_2_N_4_OS_2_	−9.011
ZINC08742427	C_23_H_19_FN_4_OS_2_	−8.938
ZINC09164985	C_23_H_19_ClN_4_OS_2_	−8.858
ZINC000008739659	C_22_H_16_F_2_N_4_OS_2_	−8.839
ZINC08856697	C_22_H_16_Cl_2_N_4_OS_2_	−8.739
ZINC17046028	C_23_H_20_N_4_OS_2_	−8.701
ZINC17159604	C_23_H_19_FN_4_OS_2_	−8.692
ZINC33258048	C_19_H_20_N_8_O_3_S_2_	−8.630
ZINC000033290624	C_25_H_31_N_5_O_2_	−8.542
ZINC000017138581	C_22_H_16_F_2_N_4_OS_2_	−8.520
ZINC000002346316	C_22_H_16_ClFN_4_OS_2_	−8.518
ZINC000008927502	C_22_H_16_ClFN_4_OS_2_	−8.286
ZINC65283170	C_20_H_23_N_7_OS	−8.256
ZINC13550820	C_23_H_19_ClN_4_O_2_S_2_	−8.203

Table 1 provides the binding affinity scores for the 4ASE–Zinc compound complexes and the reference molecule, Tivozanib. The table highlights the docking scores for the top 16 compounds, with Tivozanib, a known inhibitor.

**Table 2:** ADMET properties of the ligands and interpreting ADMET and Lipinski’s filters using the QikProp module of Schrodinger.

Compounds	Mol MW	Dipole †	#rotor	PSA	SASA	FOSA	FISA	PISA	WPSA
Tivozanib	454.869	8.613	6	108.464	754.964	271.893	129.461	286.517	67.093
ZINC000008914312	470.966	4.068	5	76.258	744.387	32.267	113.559	427.657	170.903
ZINC000008739578	487.421	6.632	5	77.517	776.043	31.78	113.492	442.425	188.346
ZINC08742427	450.548	5.579	5	76.425	760.036	119.101	114.214	427.923	98.798
ZINC09164985	467.002	5.567	5	76.419	776.446	119.786	113.926	418.314	124.419
ZINC000008739659	454.511	4.139	5	76.301	727.805	32.263	113.854	436.024	145.664
ZINC08856697	487.421	6.824	5	78.281	779.301	33.203	119.991	451.682	174.424
ZINC17046028	432.557	3.424	4	76.407	752.667	119.842	114.309	466.094	52.422
ZINC17159604	450.548	2.351	5	76.376	769.059	109.391	114.263	471.005	74.4
ZINC33258048	472.539	8.782	4	145.634	759.2	336.487	195.748	147.052	79.913
ZINC000033290624	433.552	5.637	4	88.761	789.923	445.19	94.704	250.029	0
ZINC000017138581	454.511	4.254	5	76.295	726.902	33.605	114.204	460.62	118.473
ZINC000002346316	470.966	7.198	5	78.369	753.422	31.315	113.766	454.693	153.647
ZINC000008927502	470.966	3.229	5	76.544	792.253	21.848	114.547	509.322	146.537
ZINC65283170	409.508	6.083	4	88.425	729.109	279.055	113.089	288.991	47.974
ZINC13550820	483.002	5.346	6	84.453	776.602	128.349	114.338	410.419	123.495
ZINC08913827	466.547	5.522	6	84.675	769.247	126.335	114.288	429.205	99.419

MW—molecular weight of the molecule (130.0–725.0), dipole †—computed dipole moment of the molecule (1.0–12.5), #rotor—number of non-trivial (not CX3), non-hindered (not alkene, amide, small ring) rotatable bonds (0–15), PSA—van der Waals surface area of polar nitrogen and oxygen atoms and carbonyl carbon atoms (7.0–200.0), SASA—total solvent accessible surface area in square angstroms using a probe with a 1.4 Å radius (300.0–1000.0), FOSA—hydrophobic component of the SASA (0.0–750.0), FISA—hydrophilic component of the SASA (7.0–330.0), PISA πcomponent of the SASA (0.0–450.0), WPSA—weakly polar component of the SASA (0.0–175.0).

**Table 3:** ADMET properties of the ligands and interpreting ADMET and Lipinski’s filters using the QikProp Module of Schrodinger.

Compounds	QPlogPw	QPlogPo/w	QPlogS	CIQPlogS	QPlogHERG	QPPCaco	QPlogBB
Tivozanib	13.934	3.820	−6.163	−6.420	−5.166	400.837	−1.071
ZINC000008914312	12.863	5.252	−7.345	−7.645	−5.712	593.714	−0.554
ZINC000008739578	13.265	5.591	−7.887	−7.983	−6.047	563.062	−0.564
ZINC08742427	12.943	5.106	−7.282	−7.226	−5.839	576.431	−0.763
ZINC09164985	12.916	5.384	−7.691	−7.563	−5.875	581.257	−0.714
ZINC000008739659	12.889	4.976	−6.933	−7.307	−5.661	587.572	−0.602
ZINC08856697	13.317	5.546	−7.911	−7.983	−6.103	495.654	−0.674
ZINC17046028	13.177	4.885	−6.945	−6.858	−5.988	574.101	−0.871
ZINC17159604	13.222	5.061	−7.349	−7.226	−6.19	579.808	−0.854
ZINC33258048	16.337	2.357	−5.782	−5.74	−4.453	94.666	−1.638
ZINC000033290624	13.556	4.315	−6.981	−5.727	−5.022	944.084	−0.734
ZINC000017138581	13.056	4.872	−6.794	−7.307	−5.790	581.815	−0.674
ZINC000002346316	13.347	5.270	−7.304	−7.645	−5.938	551.051	−0.618
ZINC000008927502	13.606	5.579	−8.061	−7.645	−6.544	570.418	−0.716
ZINC65283170	14.867	3.422	−5.72	−5.182	−4.994	550.325	−0.779
ZINC13550820	13.376	5.111	−7.274	−7.598	−5.796	577.1	−0.772
ZINC08913827	13.525	4.941	−7.017	−7.262	−5.864	570.856	−0.826

QPogPw—predicted water/gas partition coefficient (4.0–45.0), QPlogPo/w—predicted octanol/water partition coefficient (−2.0–6.5), QPlogS—predicted aqueous solubility, log S. S in mol dm^−3^ is the concentration of the solute in a saturated solution that is in equilibrium with the crystalline solid (−6.5–0.5), CIQPlogS—conformation-independent predicted aqueous solubility, log S. S in mol dm^−3^ is the concentration of the solute in a saturated solution that is in equilibrium with the crystalline solid (−6.5–0.5), QPlogHERG—predicted IC50 value for blockage of HERG K+ channels (concern below −5), QPPCaco—predicted apparent Caco-2 cell permeability in nm/s. Caco2 cells are a model for the gut–blood barrier. (<25 poor, >500 great), QPlogBB—predicted brain/blood partition coefficient (−3.0–1.2).

**Table 4:** The 16 compounds of interest along with the reference compound DFT calculations.

Compounds	Homo (Hartrees)	Lumo (Hartrees)	Energy Gap (ΔE)
Tivozanib	−0.216894	−0.042793	−0.174101
ZINC000008914312	−0.213681	−0.051261	−0.16242
ZINC000008739578	−0.217723	−0.05236	−0.165363
ZINC08742427	−0.209531	−0.047738	−0.161793
ZINC09164985	−0.211543	−0.048911	−0.162632
ZINC000008739659	−0.212022	−0.049825	−0.162197
ZINC08856697	−0.209859	−0.046649	−0.16321
ZINC17046028	−0.208002	−0.04429	−0.163712
ZINC17159604	−0.207501	−0.039642	−0.167859
ZINC33258048	−0.215602	−0.046696	−0.168906
ZINC000033290624	−0.194522	−0.04751	−0.147012
ZINC000017138581	−0.212671	−0.042962	−0.169709
ZINC000002346316	−0.215549	−0.051477	−0.164072
ZINC000008927502	−0.209926	−0.04891	−0.161016
ZINC65283170	−0.198739	−0.046147	−0.152592
ZINC13550820	−0.211271	−0.048374	−0.162897
ZINC08913827	−0.209046	−0.047262	−0.161784

**Table 5:** MM-GBSA calculations.

Compounds	ΔG Bind	ΔG Bind Coulomb	ΔG Bind Covalent	ΔG Bind Hbond	ΔG Bind Lipo	ΔG Bind Packing	ΔG Bind Solv GB	ΔG Bind vdW
Tivozanib	−21.95	−5.49	6.13	−0.78	−7.28	−2.73	15.7	−27.5
ZINC000008914312	−58.95	−16	1.73	−1.66	−23.01	−1.58	38.22	−56.66
ZINC000008739578	−64.75	−11.22	5.57	−1.45	−31.96	−2.05	24.45	−48.11
ZINC000017138581	−67.37	−19.8	6	−1.18	−25	−1.49	27.78	−53.68
ZINC000008927502	−69.81	−19.57	5.83	−1.14	−25.78	−1.5	28.35	−55.99

## Data Availability

Data are contained within the article and Supplementary Materials.

## Supplementary Materials

The following supporting information can be downloaded at: https://www.mdpi.com/article/10.3390/cimb46100666/s1, Figure S1. 3D representation of the redocking results for the crystallographic structure of 4ASE with its co-crystallized ligand; Figure S2. 3D representation of the redocking results for the crystallo-graphic structure of 4ASE, highlighting only the ligands without the protein; Figure S3. Molecular docking interaction diagram generated using Maestro, illustrating the interactions between 4ASE and Tivozanib; Figure S4. Molecular docking interaction diagram generated using Maestro, illus-trating the interactions between 4ASE and ZINC000002346316; Figure S5. Molecular docking in-teraction diagram generated using Maestro, illustrating the interactions between 4ASE and ZINC000008739578; Figure S6. Molecular docking interaction diagram generated using Maestro, illustrating the interactions between 4ASE and ZINC08742427; Figure S7. Molecular docking in-teraction diagram generated using Maestro, illustrating the interactions between 4ASE and ZINC08856697; Figure S8. Molecular docking interaction diagram generated using Maestro, illus-trating the interactions between 4ASE and ZINC08913827; Figure S9. Molecular docking interaction diagram generated using Maestro, illustrating the interactions between 4ASE and ZINC000008914312; Figure S10. Molecular docking interaction diagram generated using Maestro, illustrating the interactions between 4ASE and ZINC000008927502; Figure S11. Molecular docking interaction diagram generated using Maestro, illustrating the interactions between 4ASE and ZINC09164985; Figure S12. Molecular docking interaction diagram generated using Maestro, il-lustrating the interactions between 4ASE and ZINC13550820; Figure S13. Molecular docking in-teraction diagram generated using Maestro, illustrating the interactions between 4ASE and ZINC17046028; Figure S14. Molecular docking interaction diagram generated using Maestro, il-lustrating the interactions between 4ASE and ZINC000017138581; Figure S15. Molecular docking interaction diagram generated using Maestro, illustrating the interactions between 4ASE and ZINC17159604; Figure S16. Molecular docking interaction diagram generated using Maestro, il-lustrating the interactions between 4ASE and ZINC33258048; Figure S17. Molecular docking in-teraction diagram generated using Maestro, illustrating the interactions between 4ASE and ZINC65283170; Figure S18. Histogram of protein-ligand contacts from molecular dynamics (MD) simulations for 4ASE in complex with Tivozanib; Figure S19. Histogram of protein-ligand contacts from molecular dynamics (MD) simulations for 4ASE in complex with ZINC000008739578; Figure S20. Histogram of protein-ligand contacts from molecular dynamics (MD) simulations for 4ASE in complex with ZINC000008914312; Figure S21. Histogram of protein-ligand contacts from molec-ular dynamics (MD) simulations for 4ASE in complex with ZINC000008927502; Figure S22. His-togram of protein-ligand contacts from molecular dynamics (MD) simulations for 4ASE in complex with ZINC000017138581.
