# Supplementary material for: In Silico Screening of 1,3,4-Thiadiazole Derivatives as Inhibitors of Vascular Endothelial Growth Factor Receptor-2 (VEGFR-2)
Source: Curr Issues Mol Biol. 2024 Oct 6;46(10):11220–35. doi: 10.3390/cimb46100666 (PMC11505934; doi:10.3390/cimb46100666)
Supplement: Supplementary file 1 [file cimb-46-00666-s001.zip › cimb-3192541-supplementary.pdf]

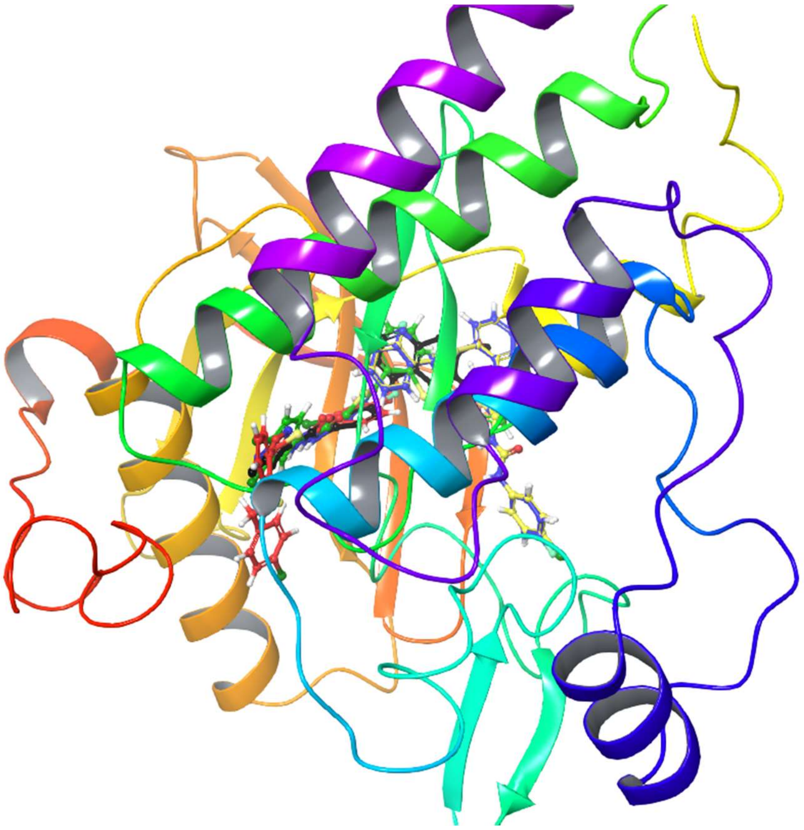

Figure S1. 3D representation of the redocking results for the crystallographic structure of 4ASE with its co-crystallized ligand, Tivozanib (black). The binding poses of the additional ligands—ZINC000008914312 (red), ZINC000008739578 (green), ZINC000008927502 (blue), and ZINC000017138581 (yellow)—are shown overlapping within the active site.

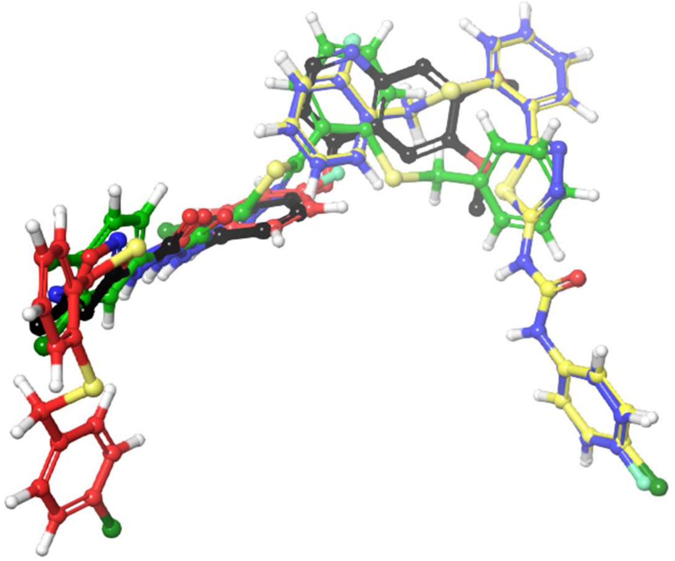

Figure S2. 3D representation of the redocking results for the crystallographic structure of 4ASE, highlighting only the ligands without the protein. The co-crystallized ligand, Tivozanib (black), is shown along with the binding poses of the additional ligands—ZINC000008914312 (red), ZINC000008739578 (green), ZINC000008927502 (blue), and ZINC000017138581 (yellow)—all overlapping within the active site.

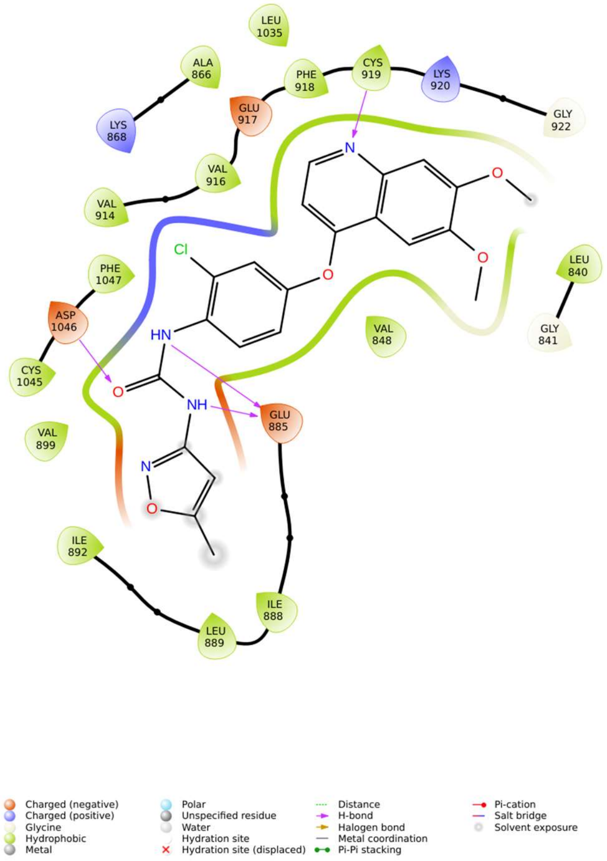

Figure S3. Molecular docking interaction diagram generated using Maestro, illustrating the interactions between 4ASE and Tivozanib.

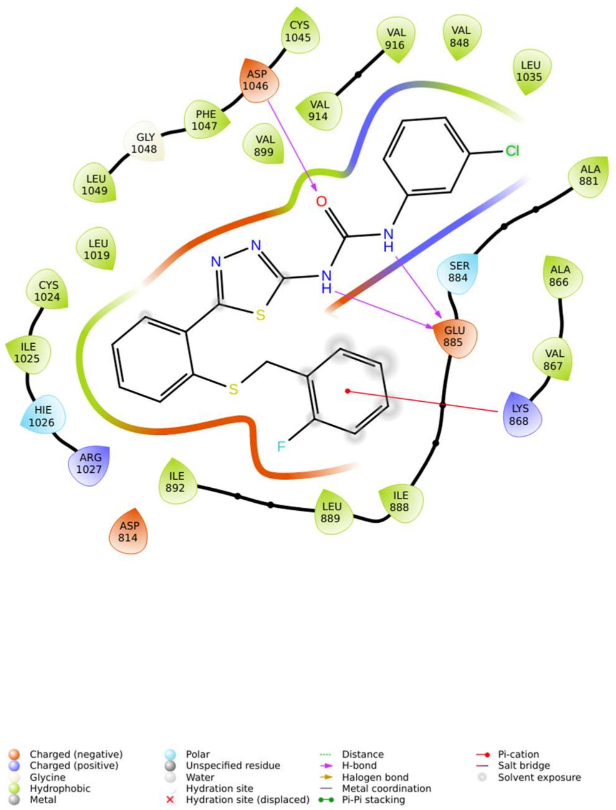

Figure S4. Molecular docking interaction diagram generated using Maestro, illustrating the interactions between 4ASE and ZINC000002346316.

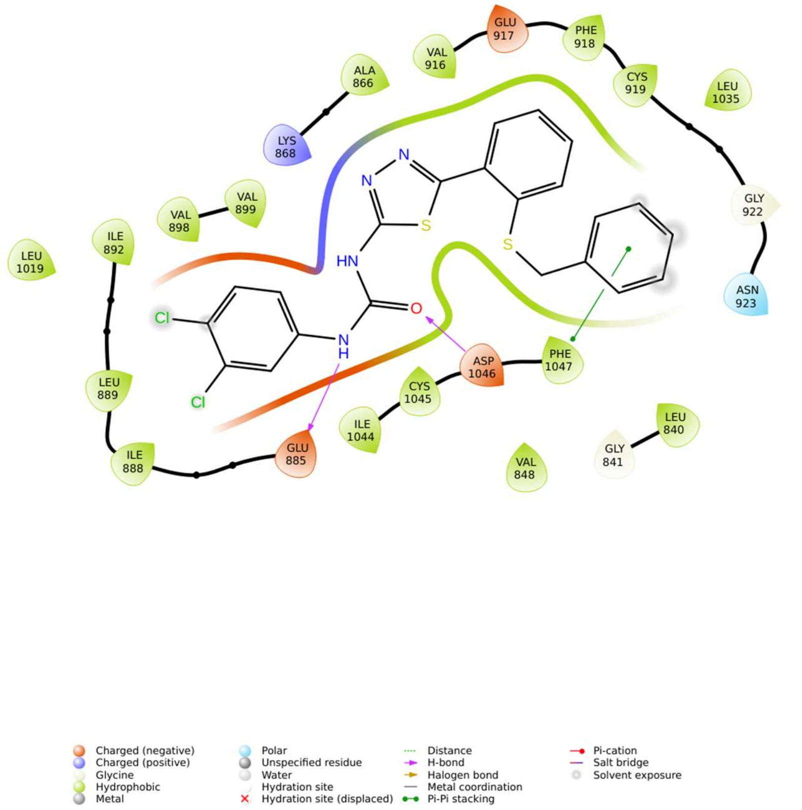

Figure S5. Molecular docking interaction diagram generated using Maestro, illustrating the interactions between 4ASE and ZINC000008739578.

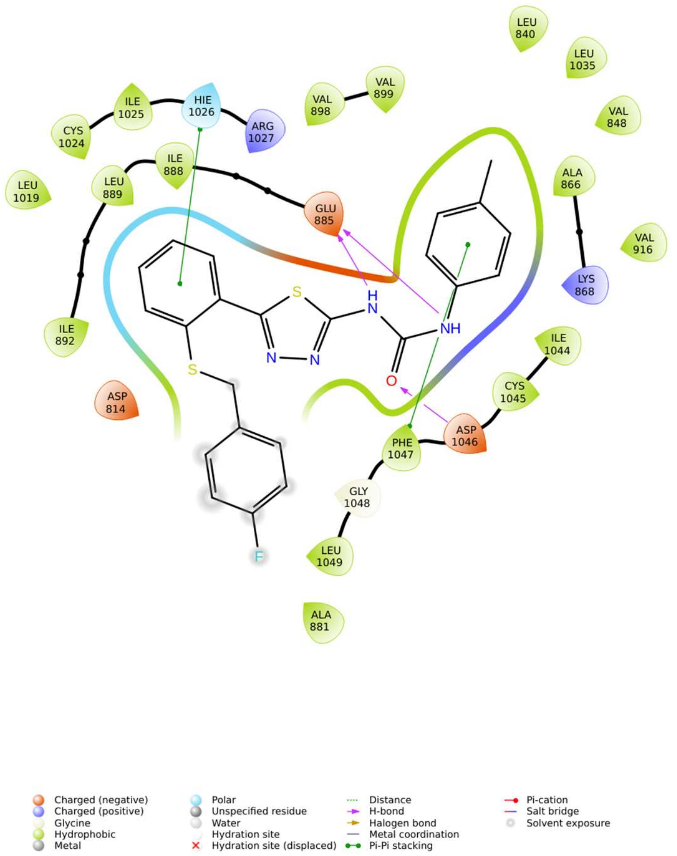

Figure S6. Molecular docking interaction diagram generated using Maestro, illustrating the interactions between 4ASE and ZINC08742427

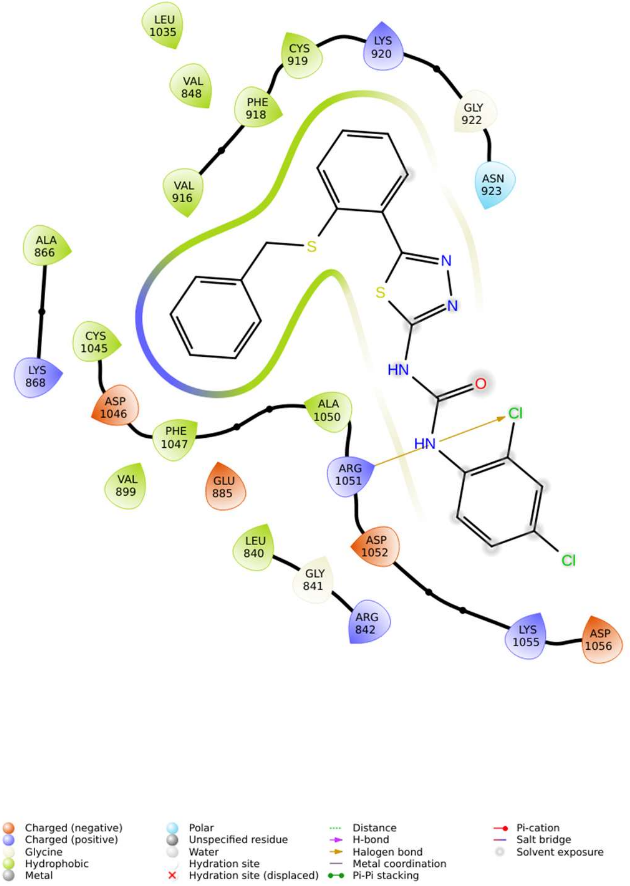

Figure S7. Molecular docking interaction diagram generated using Maestro, illustrating the interactions between 4ASE and ZINC08856697

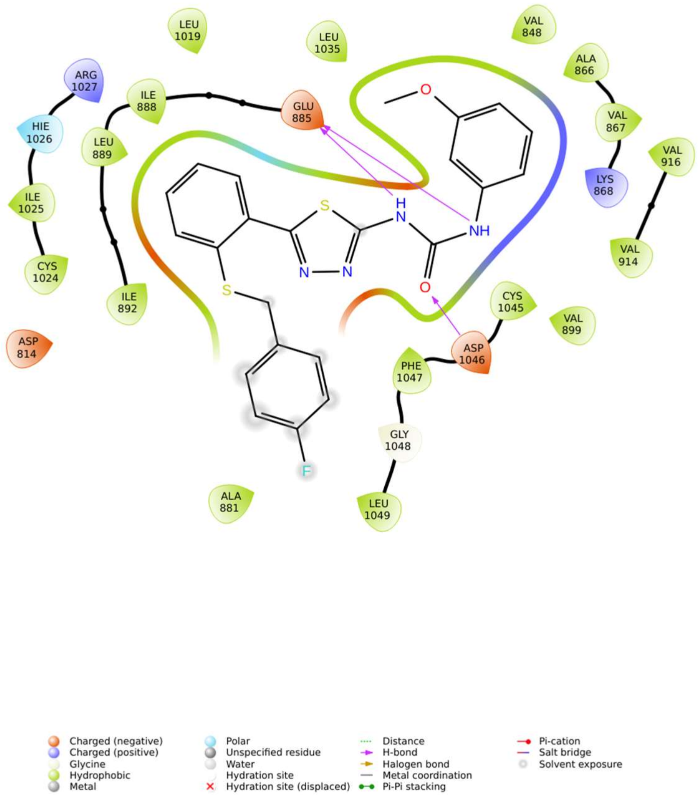

Figure S8. Molecular docking interaction diagram generated using Maestro, illustrating the interactions between 4ASE and ZINC08913827

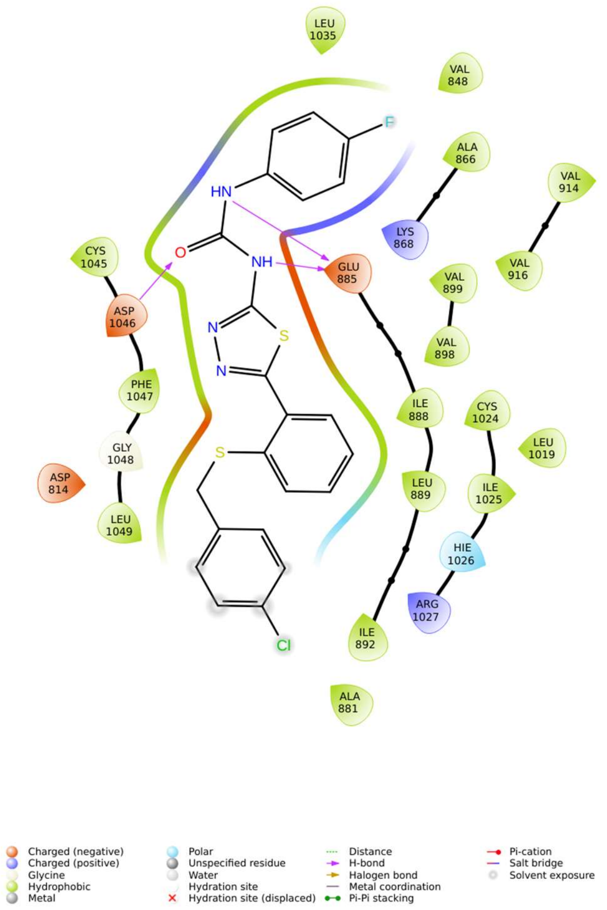

Figure S9. Molecular docking interaction diagram generated using Maestro, illustrating the interactions between 4ASE and ZINC000008914312

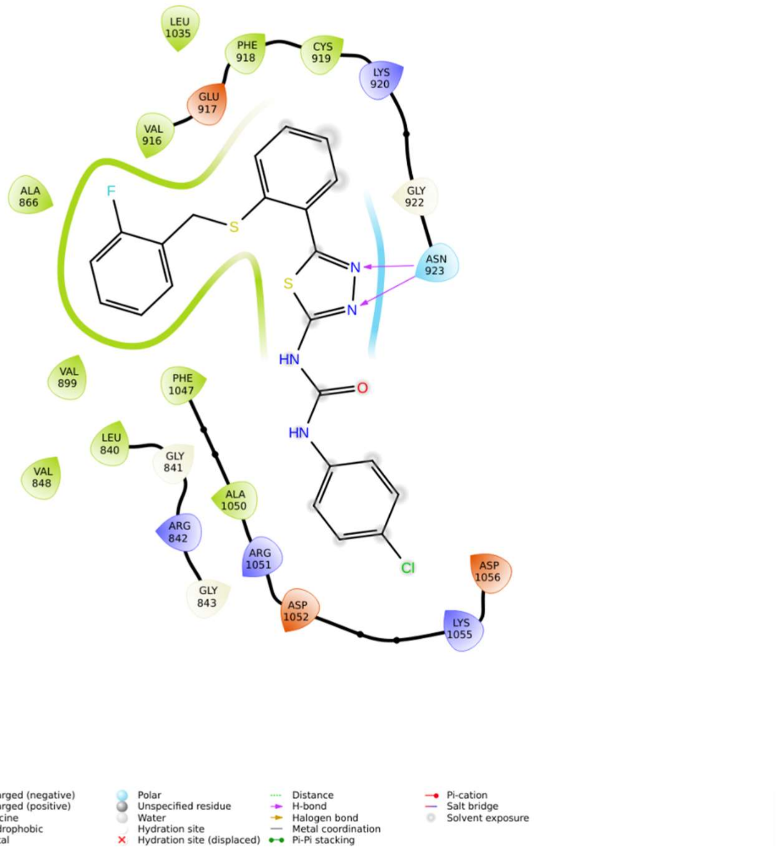

Figure S10. Molecular docking interaction diagram generated using Maestro, illustrating the interactions between 4ASE and ZINC000008927502.

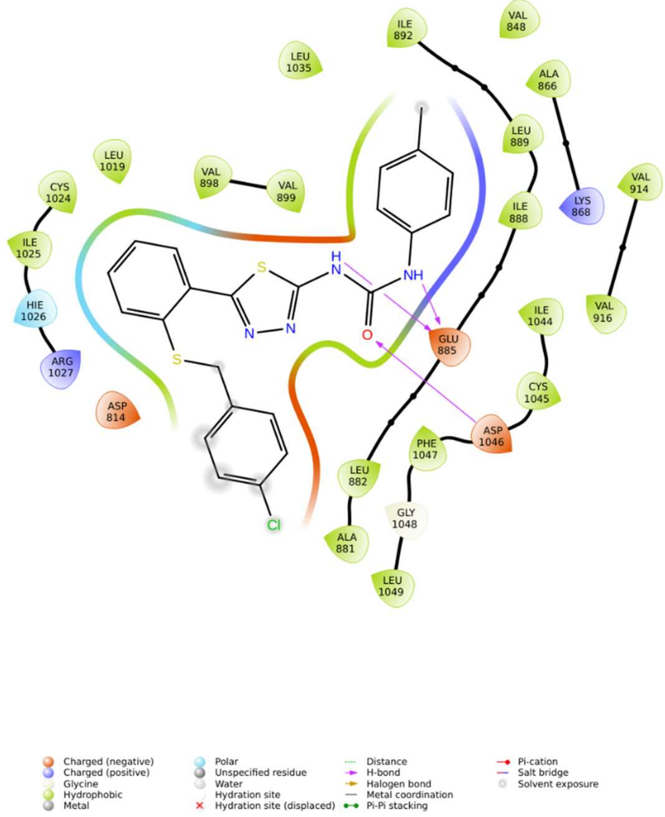

Figure S11. Molecular docking interaction diagram generated using Maestro, illustrating the interactions between 4ASE and ZINC09164985

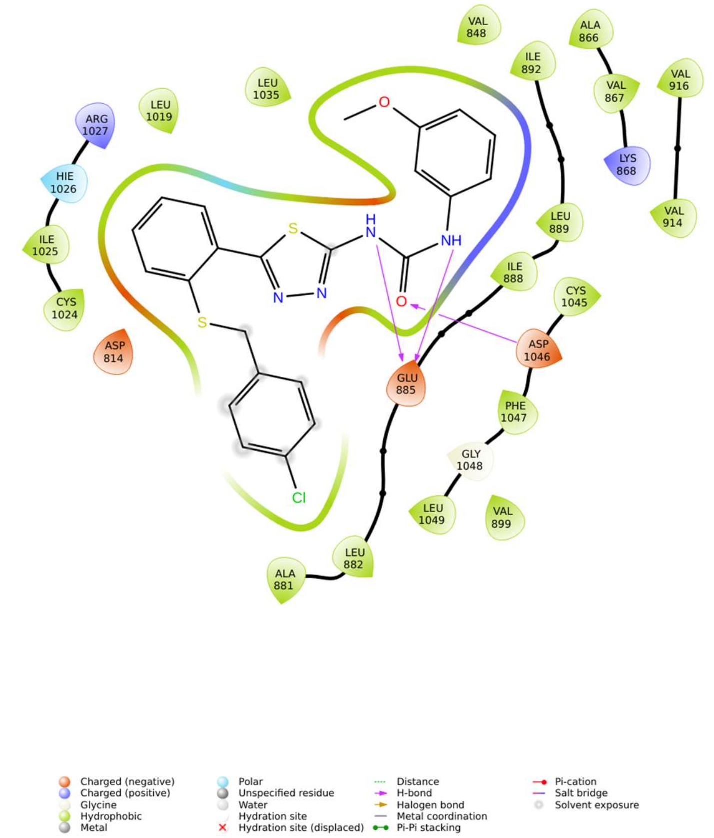

Figure S12. Molecular docking interaction diagram generated using Maestro, illustrating the interactions between 4ASE and ZINC13550820

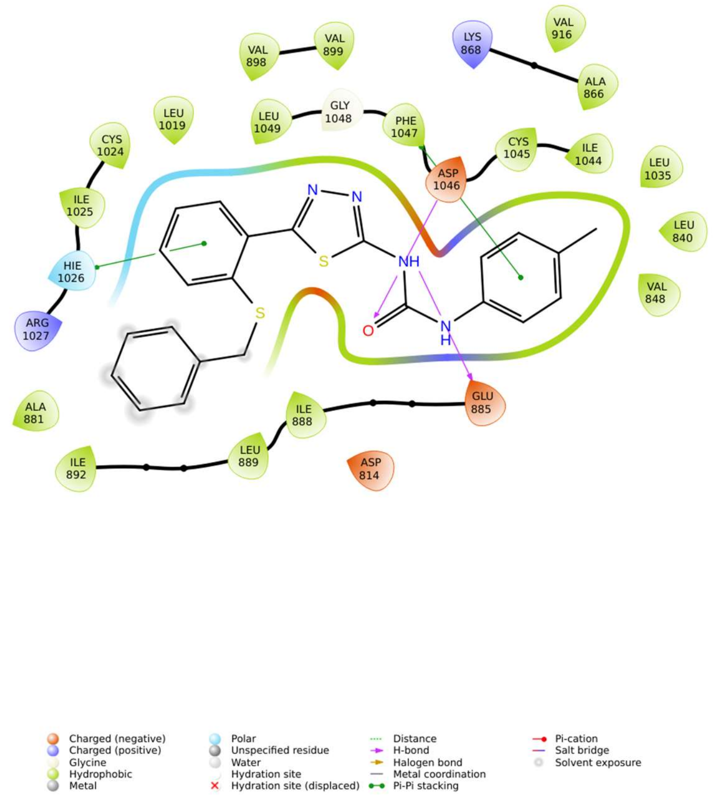

Figure S13. Molecular docking interaction diagram generated using Maestro, illustrating the interactions between 4ASE and ZINC17046028

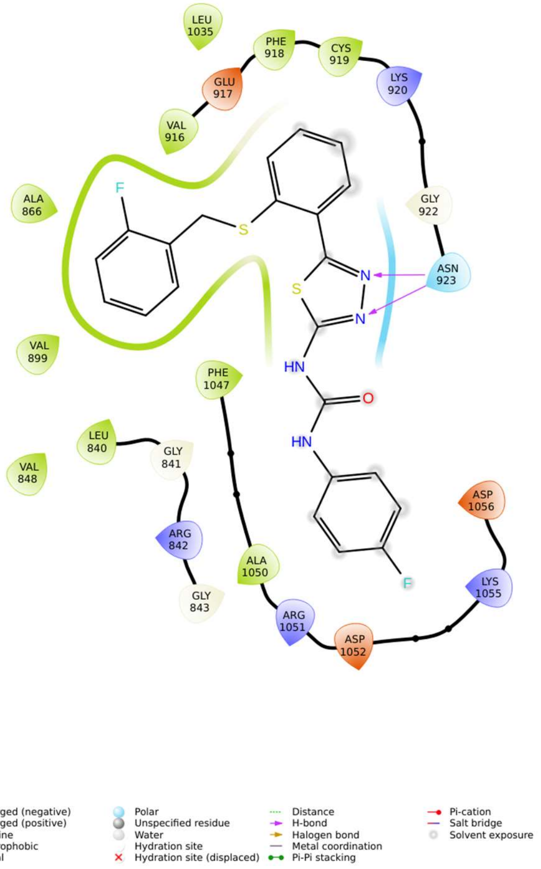

Figure S14. Molecular docking interaction diagram generated using Maestro, illustrating the interactions between 4ASE and ZINC000017138581

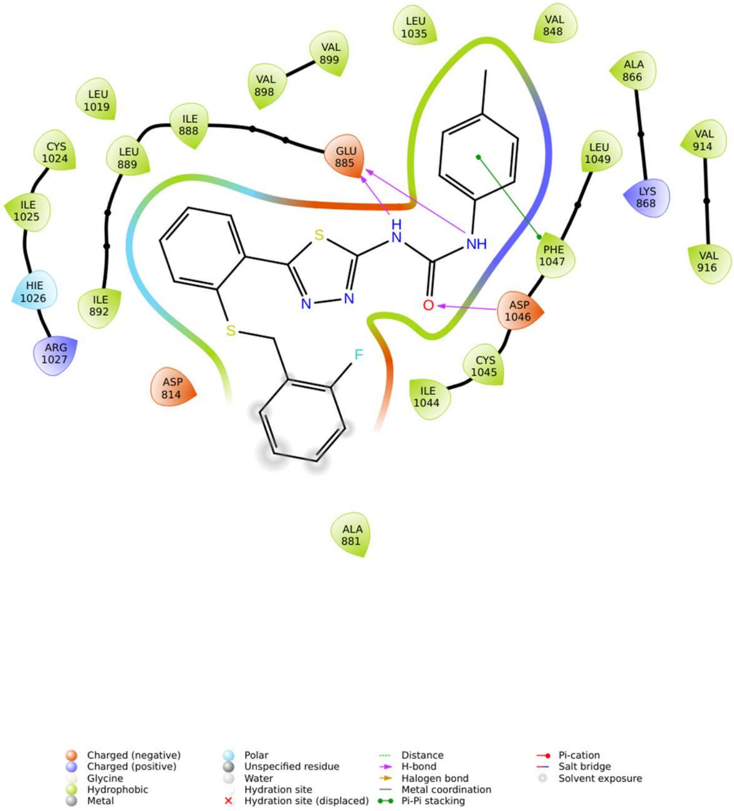

Figure S15. Molecular docking interaction diagram generated using Maestro, illustrating the interactions between 4ASE and ZINC17159604

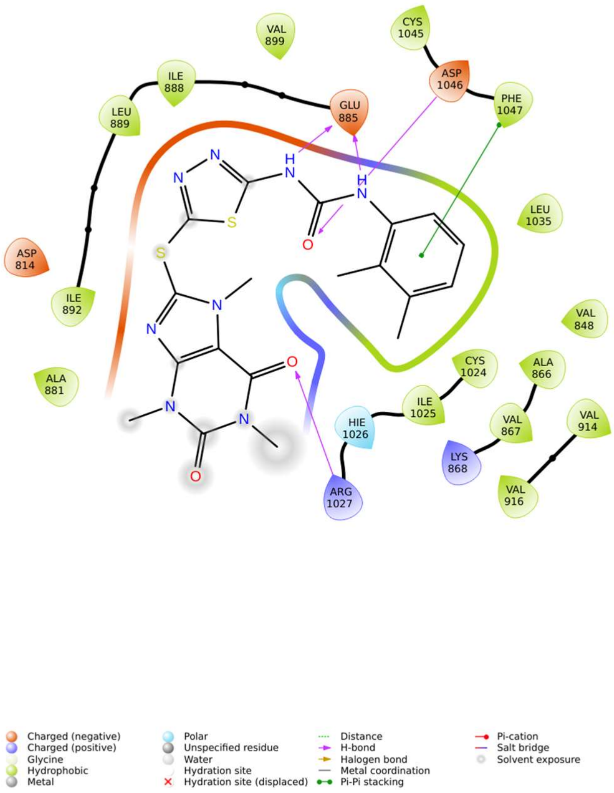

Figure S16. Molecular docking interaction diagram generated using Maestro, illustrating the interactions between 4ASE and ZINC33258048

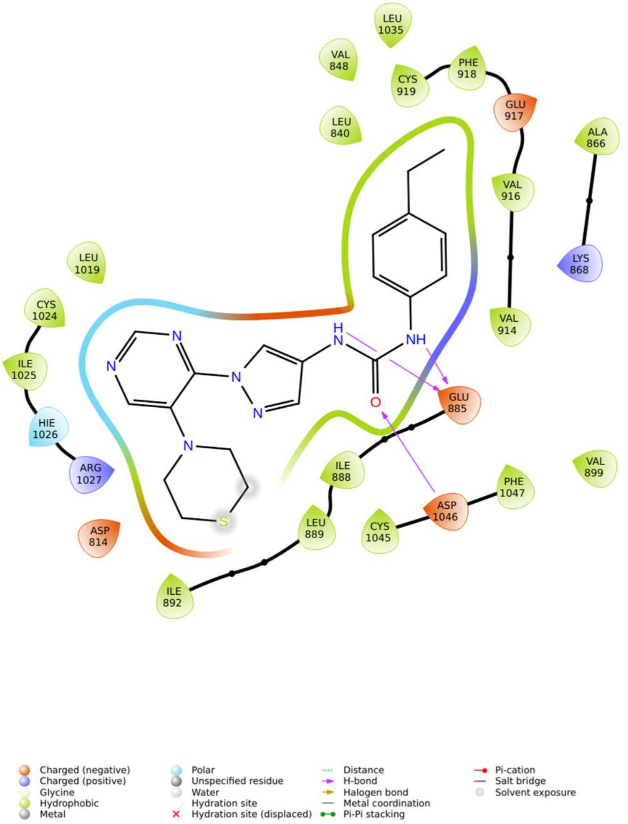

Figure S17. Molecular docking interaction diagram generated using Maestro, illustrating the interactions between 4ASE and ZINC65283170

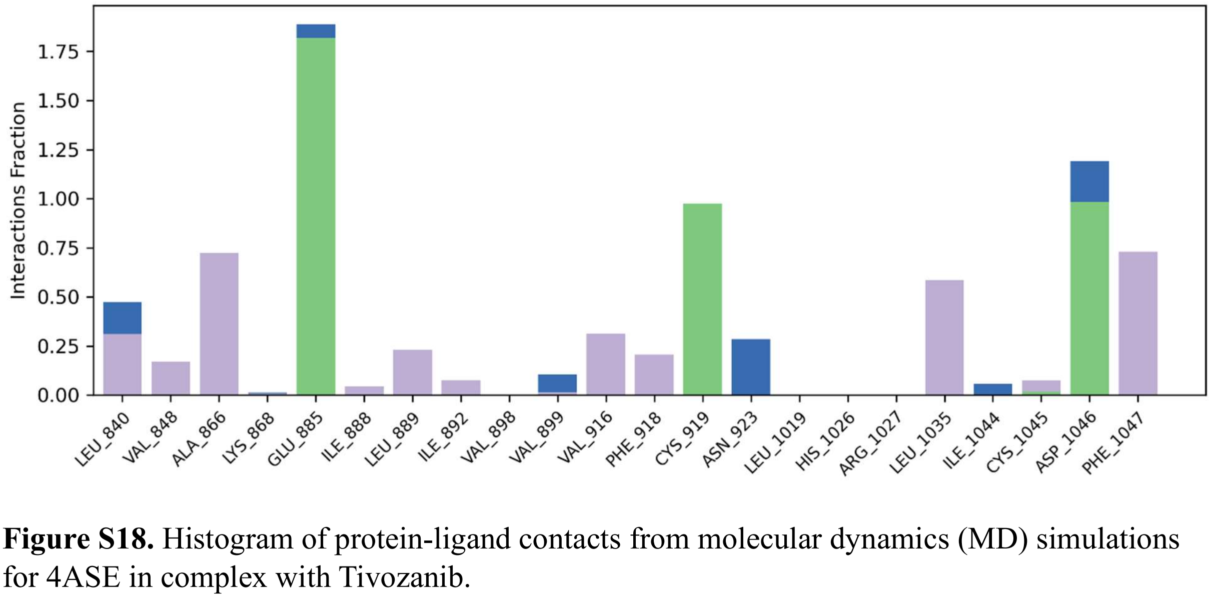

Figure S18. Histogram of protein-ligand contacts from molecular dynamics (MD) simulations for 4ASE in complex with Tivozanib.

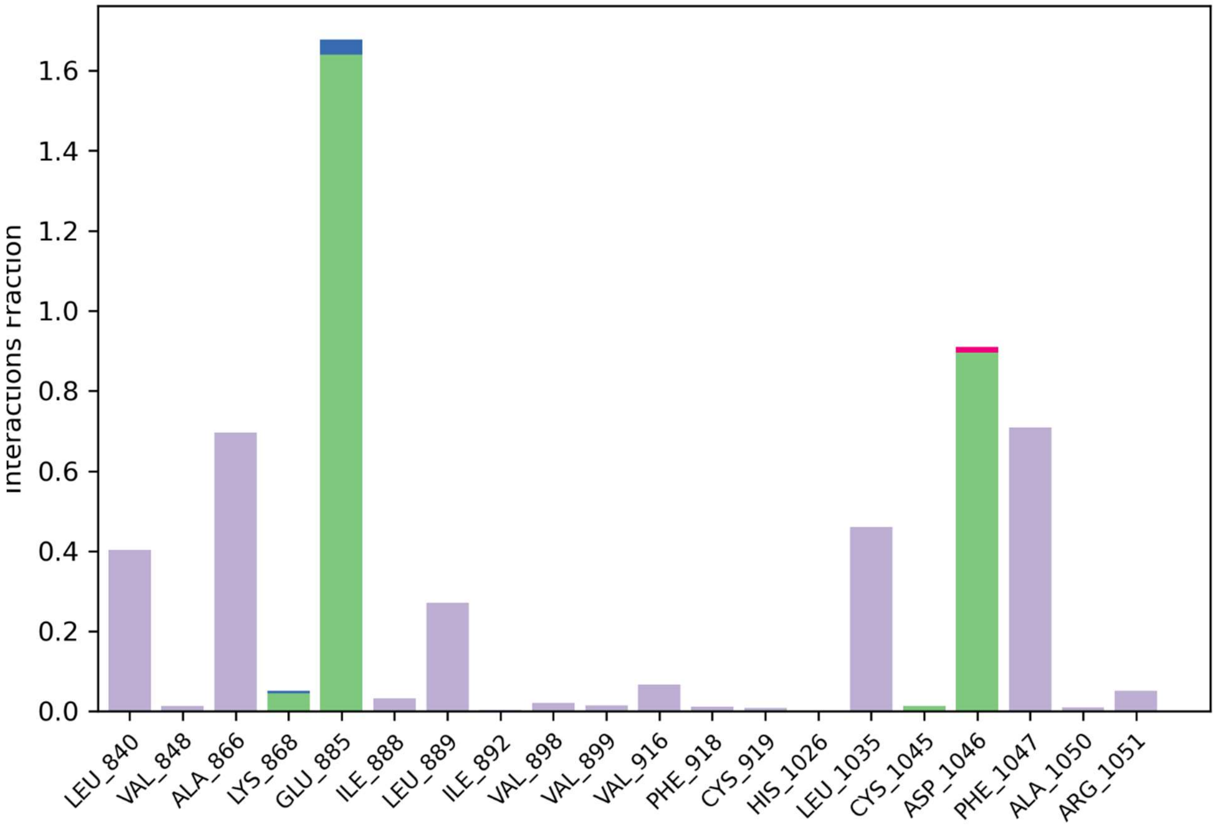

Figure S19. Histogram of protein-ligand contacts from molecular dynamics (MD) simulations for 4ASE in complex with ZINC000008739578.

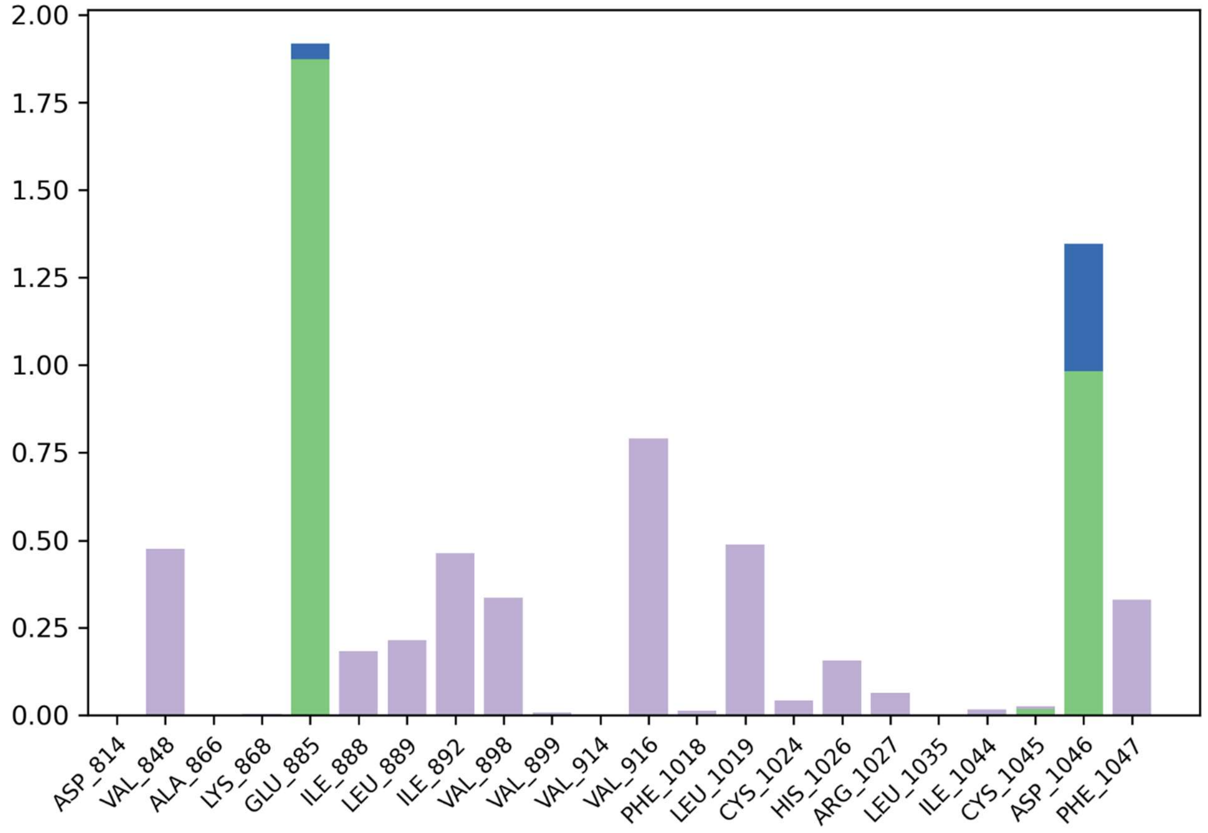

Figure S20. Histogram of protein-ligand contacts from molecular dynamics (MD) simulations for 4ASE in complex with ZINC000008914312

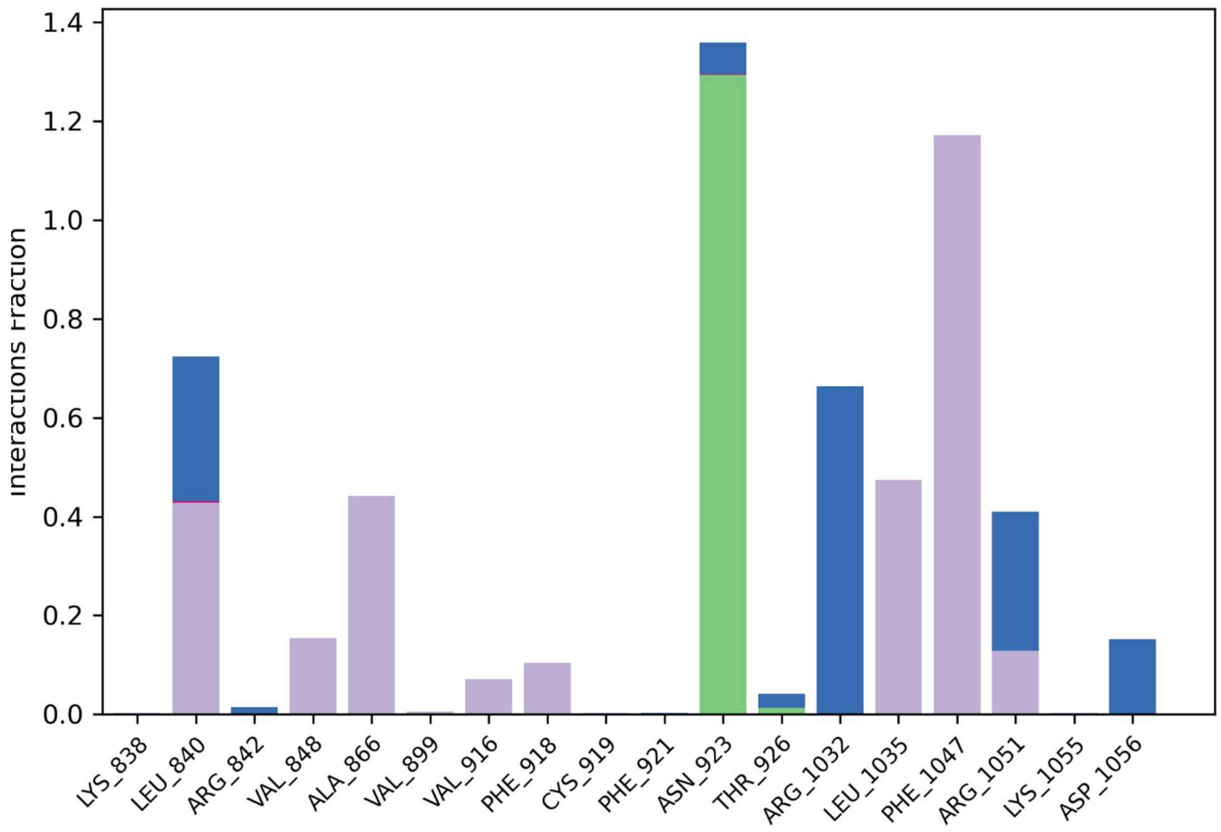

Figure S21. Histogram of protein-ligand contacts from molecular dynamics (MD) simulations for 4ASE in complex with ZINC000008927502

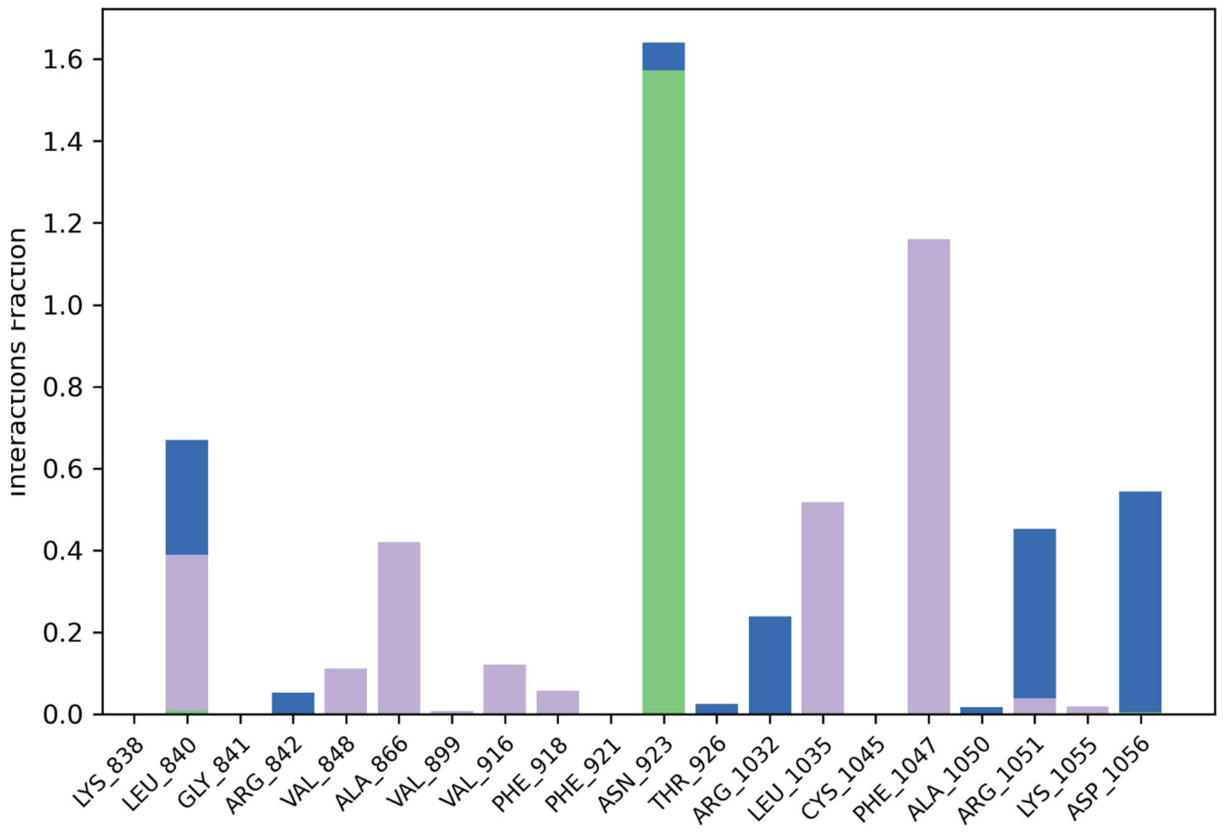

Figure S22. Histogram of protein-ligand contacts from molecular dynamics (MD) simulations for 4ASE in complex with ZINC000017138581
